# Preparation and Properties of Lightweight Aggregates from Discarded Al_2_O_3_-ZrO_2_-C Refractories

**DOI:** 10.3390/ma17163968

**Published:** 2024-08-09

**Authors:** Shuli Sun, Junfeng Qu, Mengyong Sun, Xinming Ren, Cheng Gong, Xin Mu, Wenyu Zan, Zhangyan Zhou, Chengji Deng, Beiyue Ma

**Affiliations:** 1 Key Laboratory for Ecological Metallurgy of Multimetallic Mineral, Ministry of Education, Northeastern University, Shenyang 110819, China; sunsl1124@outlook.com (S.S.); maby2005@126.com (C.G.); maby@smm.neu.edu.cn (X.M.); a18738680638@126.com (W.Z.); zzy1109812710@163.com (Z.Z.); 2 Inner Mongolia Metal Material Research Institute, Yantai 264003, China; armytwo@163.com (J.Q.); sunmy@mail.nwpu.edu.cn (M.S.); renxm1124@163.com (X.R.); 3 The State Key Laboratory of Refractories and Metallurgy, Wuhan University of Science and Technology, Wuhan 430081, China; cjdeng@wust.edu.cn

**Keywords:** used refractories, magnesia, lightweight aggregates, calcined temperature, thermal shock resistance

## Abstract

Refractory materials are an important pillar for the stable development of the high-temperature industry. A large amount of waste refractories needs to be further disposed of every year, so it is of great significance to carry out research on the recycling of used refractories. In this work, lightweight composite aggregate was prepared by using discarded Al_2_O_3_-ZrO_2_-C refractories as the main raw material, and the performance of the prepared lightweight aggregate was improved by adjusting the calcination temperature and introducing light calcined magnesia additives. The results showed that the cold compressive strength and thermal shock resistance of the lightweight aggregates were significantly improved with increasing calcination temperature. Moreover, the introduction of light calcined magnesia can effectively improve the apparent porosity, cold compressive strength, and thermal shock resistance of the prepared lightweight aggregates at the calcination temperature of 1400 °C. Consequently, this work provides a useful reference for the resource utilization of used refractories, while the prepared lightweight aggregates are expected to be applied in the field of high-temperature insulation.

## 1. Introduction

Since the beginning of the 21st century, the global metallurgical industry has been booming. The refractory industry, as its upstream support, has also developed rapidly. Therefore, it is inevitable that a large amount of metallurgical solid waste and refractory solid waste will be generated every year. In comparison, metallurgical solid waste has been studied in depth and fully utilized [1,2,3]. If these millions of tons of used refractories that are randomly discarded and buried can be recycled, it will not only save a lot of resources but also play a very important role in improving and protecting the environment. At the same time, recycling refractories after use can not only improve the economic benefits of relevant enterprises but also have good social effects [4]. The current society is facing the problem of a shortage of mineral resources. The recycling and reuse of used refractories is a general trend and an inevitable requirement to maintain the sustainable development of the refractory industry. This strategy is a win-win situation for refractory material manufacturers and users. Overall, there are still several problems in the utilization of waste refractories. One of them is the low comprehensive utilization rate and the large gap between the utilization rates of different countries; the other is the low added value, such as being directly used as an additive after crushing or downgraded as a raw material [5]. Undoubtedly, the rationalization of waste refractories still needs to continue to invest more attention and research.

The lightweighting of the working lining of industrial furnaces is an important research direction of refractory materials: on the one hand, the lightweight refractories for working linings can effectively reduce the heat dissipation of the industrial furnace; on the other hand, because lightweight refractory materials have a porous structure, they can effectively alleviate the thermal stress during drastic temperature changes, thus improving the high-temperature stability of the materials [6,7,8]. However, porous materials with good thermal conductivity usually have poor mechanical properties. Lightweight aggregates, due to their special structure and simple preparation, are the most promising candidate for energy-saving refractory materials. There are various methods for preparing lightweight aggregates, such as the pore-forming agent method [2], foaming method [9], and in situ decomposition technique [10]. As with other refractories, lightweight aggregates are generally made from processed ores or synthetic chemical materials. In recent years, in response to the global development strategy of energy conservation and emission reduction, researchers have investigated the application potential of zero-cost materials such as tailings [11], industrial solid waste [12], and domestic waste [13] as raw materials. Therefore, for a long time to come, the reuse of solid waste in lightweight aggregates will be one of the points of research emphasis and hotspots worthy of attention.

Al_2_O_3_-ZrO_2_-C refractories are widely used in continuous casting systems for steelmaking, such as long nozzle, submerged entry nozzle, integral stopper, and sliding plate, because of their excellent high-temperature stability, slag resistance, and long service life [14,15,16]. However, with its widespread application and massive use, how to dispose of Al_2_O_3_-ZrO_2_-C refractories after use has become an urgent problem to be solved. Under this background, the aim of this paper is to utilize the Al_2_O_3_-ZrO_2_-C refractories to prepare lightweight aggregates with balanced properties and wide applications. The effects of the calcination temperature on the phase composition, microstructure, porosity, and mechanical properties of the lightweight aggregates were specifically studied, and moreover, the feasibility of introducing light calcined magnesia to further improve the specific properties of the prepared lightweight aggregates was investigated.

## 2. Experimental Section

### 2.1. Raw Materials

Used Al_2_O_3_-ZrO_2_-C refractories (Yingkou, China) were selected as the starting raw materials and light calcined magnesia (Yingkou, China) was selected as the additive. Their chemical compositions are shown in Table 1. 

### 2.2. Processing

In the first step, the received used Al_2_O_3_-ZrO_2_-C refractories were crushed and then sieved with a 100-mesh sieve; in the second step, the raw materials and additives (light calcined magnesia, ≤74 μm) were weighed separately as required (100:0, 70:30, 60:40, and 50:50), and then they were ball-milled well with a rotation speed of 200 r·min−1 for 2 h; in the third step, the mixed powders were pressed into the cylindrical green body of Φ16 mm × 16 mm with a molding pressure of 50 MPa for 3 min; and in the fourth step, the different green bodies were placed in a muffle furnace at 1200, 1300, and 1400 °C for 1 h.

### 2.3. Characterization

X-ray diffraction technology (XRD, D8, Bruker, Karlsruhe, Germany) was used to detect the phase composition of the calcined samples, and the Bragg angle (*θ*) range was 10° to 90°. Scanning electron microscopy technology (SEM, S4800, Hitachi, Tokyo, Japan) and energy dispersive X-ray spectrometry technology (EDS, Ultim Max, Oxford Instruments, Oxford, England) were used to record the microstructure and the micro-area elements of the calcined samples. The vacuum method (testing parameters: distilled water medium, vacuum degree < 2.5 kPa) was chosen to measure the apparent porosity and bulk density of the calcined samples. The uniaxial compression method (WDW-100, Shandong Wanchen Testing Machine Co., Ltd., Jinan, China; loading speed of 0.5 mm·min^−1^) was chosen to measure the cold compressive strength of the calcined samples. The compressed air quenching method (recording of the cold temperature compressive strength after five quenchings) was chosen to measure the thermal shock resistance of the calcined samples [17].

## 3. Results and Analysis

### 3.1. Determination of Calcination Temperature

#### 3.1.1. Phase Composition

**Figure 1:**
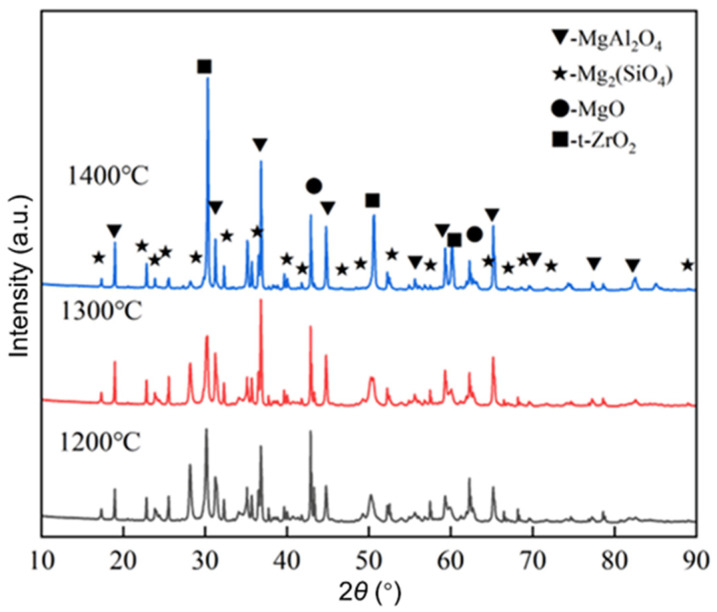
XRD patterns of the sample with 40% of magnesia additive after calcining at 1200, 1300, and 1400 °C for 1 h.

Figure 1 shows the XRD patterns of the sample with 40% of magnesia additive after calcining at 1200 °C, 1300 °C, and 1400 °C for 1 h. From Figure 1, the phase composition of the prepared lightweight aggregate samples at different calcination temperatures is composed of MgAl_2_O_4_, Mg_2_SiO_4_, MgO, and t-ZrO_2_; meanwhile, the relative intensity of the diffraction peaks of the MgAl_2_O_4_ phase increases with rising temperature, which implies that the increase in calcination temperature will promote the reaction of alumina (from used Al_2_O_3_-C refractories) and light calcined magnesia. In addition, by comparing the relative intensities of the diffraction peaks of MgAl_2_O_4_ and Mg_2_SiO_4_ phases, the content of the MgAl_2_O_4_ phase is higher than that of the Mg_2_SiO_4_ phase.

#### 3.1.2. Microstructure Evolution

**Figure 2:**
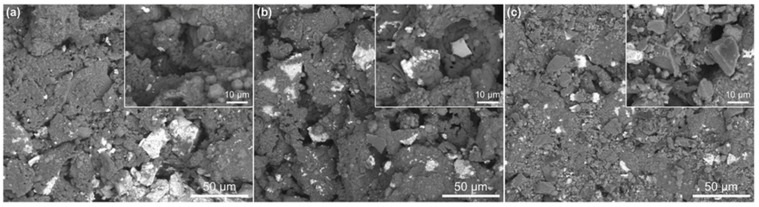
SEM images of the sample with 40% of magnesia additive after calcining at 1200 °C (**a**), 1300 °C (**b**), and 1400 °C (**c**) for 1 h.

Figure 2 presents the SEM images of the sample with 40% of magnesia additive after calcining at 1200 °C, 1300 °C, and 1400 °C for 1 h. From Figure 2a, the gaps between the particles of the sample calcined at 1200 °C are larger and there are more pores, so only a small number of particles reacted with each other and formed the new MgAl_2_O_4_ phase at this calcination temperature. As the calcination temperature increases to 1300 °C (Figure 2b), the particles of the prepared lightweight aggregate sample are tightly combined, and the large pores gradually disappear. As shown in Figure 2c, when the temperature is set at 1400 °C, in addition to the tighter bonding between particles, the prepared sample also forms more of the MgAl_2_O_4_ phase.

#### 3.1.3. Porosity and Density

**Figure 3:**
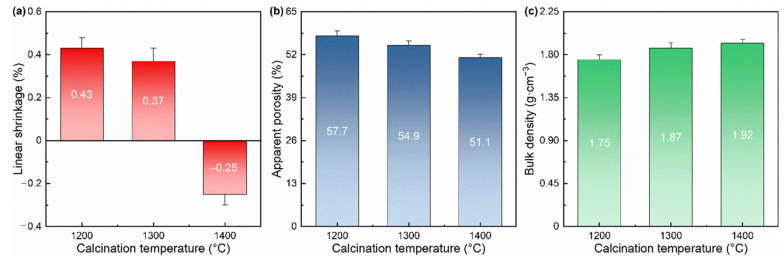
(**a**) Linear shrinkage, (**b**) apparent porosity, and (**c**) bulk density of the samples with 40% of magnesia additive after calcining at 1200 °C, 1300 °C, and 1400 °C for 1 h.

Figure 3 shows the linear shrinkage, apparent porosity, and bulk density of the samples with 40% of magnesia additive after calcining at 1200, 1300, and 1400 for 1 h. From Figure 3a, as the calcination temperature increases, the linear shrinkage of the sample decreases slightly at first (from 0.43% at 1200 °C to 0.37% at 1300 °C) and then decreases sharply. The linear rate of change value becomes negative at 1400 °C, which means that the volume shrinkage due to sintering exceeds the volume expansion due to reaction. The linear shrinkage value becomes negative at 1400 °C (−0.25%), which means that the volume shrinkage due to sintering exceeds the volume expansion due to reaction (theoretically, the reaction of alumina and magnesium oxide to form magnesium-alumina spinel produces a volume expansion of ~8% [18]). 

As shown in Figure 3b, the apparent porosity of samples prepared at higher calcination temperatures is smaller, specifically decreasing from 57.7% at 1200 °C to 51.1% at 1400 °C. The reasons why all samples show high porosity (>50%) are as follows: the graphite in the raw materials acts as a pore-forming agent; the volume expansion effect occurs during the reaction of alumina (from used Al_2_O_3_-ZrO_2_-C refractories) and magnesia (from the additive) to form magnesia-alumina spinel. From Figure 3c, the changing trend of the bulk density of the samples is exactly opposite to that of their apparent porosity. As the calcination temperature increases, the bulk density of the sample increases accordingly, but the overall change is not significant.

#### 3.1.4. Cold Compressive Strength and Thermal Shock Resistance

**Figure 4:**
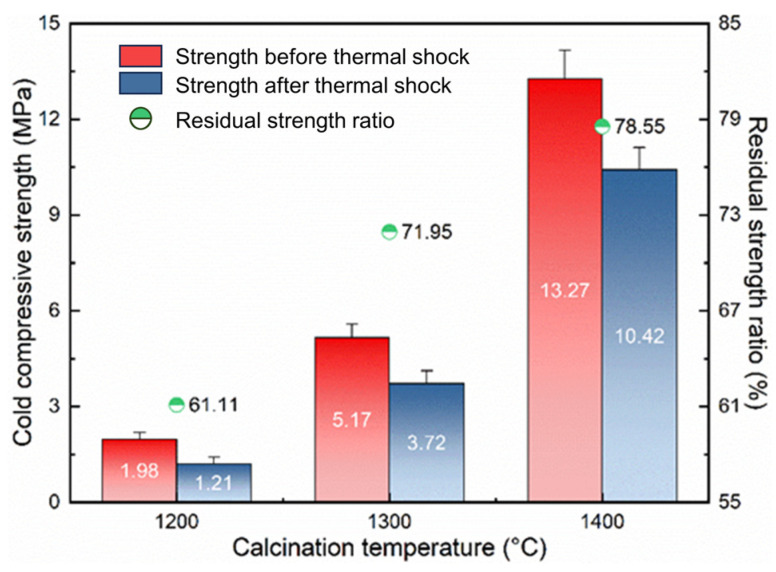
Cold compressive strength and thermal shock resistance of the samples with 40% of magnesia additive after calcining at 1200 °C, 1300 °C, and 1400 °C for 1 h.

Figure 4 shows the cold compressive strength and thermal shock resistance of the samples of magnesia additive after calcining at 1200 °C, 1300 °C, and 1400 °C for 1 h. For lightweight refractory aggregates, in addition to a smaller density, they also need to have sufficient strength and excellent high-temperature stability [8,19]. From Figure 4, the cold compressive strength of the samples is increased with rising calcination temperature, especially from 1300 °C to 1400 °C. On the one hand, the microstructure of the samples prepared at higher calcination temperatures is denser; on the other hand, the samples prepared at higher calcination temperatures contain more of the MgAl_2_O_4_ phase. Therefore, the sample calcined at 1400 °C shows the best mechanical properties, with the cold compressive strength of 13.27 MPa. In order to characterize the high-temperature stability performance of the samples, the cold compressive strength of the samples after completing three quenchings was recorded. As shown in Figure 4, after the quenching experiment, the strength of the samples is decreased. By comparing the calculated residual strength ratio, the thermal shock resistance of the samples enhances with increasing calcination temperature. Among them, the residual strength ratio of the sample calcined at 1400 °C is increased to 78.55%. This beneficial result can be attributed to the different phase contents of the samples. The sample prepared at 1400 °C contains more of the MgAl_2_O_4_ phase, which can effectively improve the thermal shock resistance of the sample because of its lower thermal expansion coefficient [20].

According to the above results, the comprehensive performance of the sample calcined at 1400 °C is relatively moderate, so this temperature is chosen to continue the follow-up study.

### 3.2. Effect of Light Calcined Magnesia Addition

#### 3.2.1. Phase Composition

**Figure 5:**
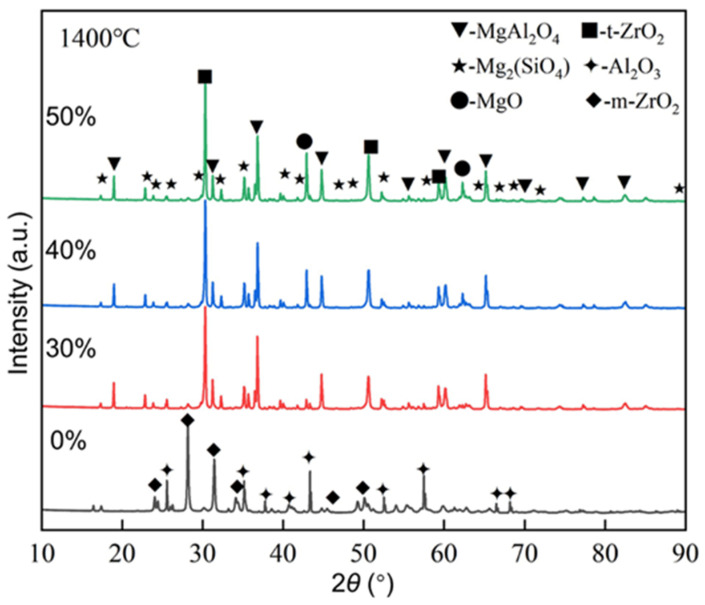
XRD patterns of the samples with different amounts of light calcined magnesia after calcining at 1400 °C for 1 h.

Figure 5 shows the XRD patterns of the samples with different amounts of light calcined magnesia after calcining at 1400 °C for 1 h. From Figure 5, the phase composition of the prepared lightweight aggregate samples is obviously different with the different addition amounts of light calcined magnesia. For the blank sample, it only contains m-ZrO_2_ and Al_2_O_3_ phases. With the introduction of additives, the phase composition of the samples changes to MgAl_2_O_4_, Mg_2_SiO_4_, MgO, and t-ZrO_2_ phases. This is because MgO and ZrO_2_ can form a substitutional solid solution (ZrO_2_(MgO)ss), allowing ZrO2 to maintain a tetragonal crystal structure at low temperatures [21]. In addition, by comparing the diffraction peak intensities of different phases, the MgAl_2_O_4_ content is much greater than the Mg_2_SiO_4_ content.

#### 3.2.2. Microstructure Evolution

**Figure 6:**
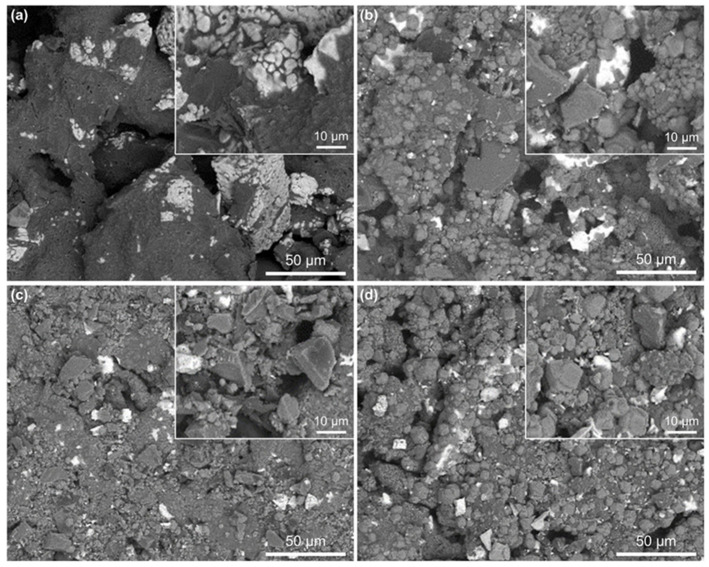
SEM images of the samples with different amounts of light calcined magnesia after cal-cining at 1400 °C for 1 h. (**a**) 0%; (**b**) 30%; (**c**) 40%; (**d**) 50%.

Figure 6 presents the SEM images of the samples with different amounts of light calcined magnesia after firing at 1400 °C for 1 h. The micromorphology of the blank sample without light calcined magnesia is shown in Figure 6a; the larger particles are not obviously combined with each other, exhibiting an overall loose microstructure. On the contrary, for the samples containing light calcined magnesia, their particle size gradually decreases, and good interfacial bonding between the particles is formed due to the occurrence of chemical reactions. Meanwhile, the pore size of the samples gradually decreases, but the number gradually increases.

#### 3.2.3. Porosity and Density

**Figure 7:**
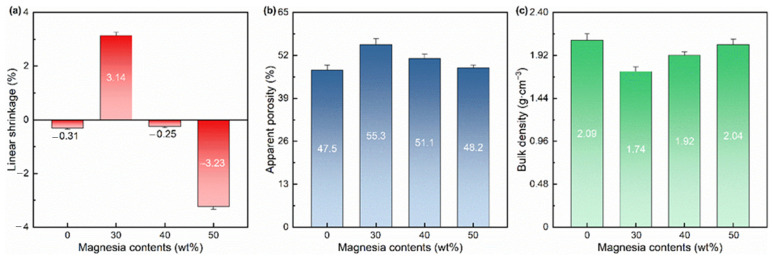
(**a**) Linear shrinkage, (**b**) apparent porosity, and (**c**) bulk density of the samples of the samples with different amounts of light calcined magnesia after calcining at 1400 °C for 1 h.

Figure 7 shows the apparent porosity and bulk density of the samples with different amounts of light calcined magnesia after firing at 1400 °C for 1 h. From Figure 7a, compared with the blank sample (−0.31%), the linear shrinkage of the additive-containing samples increases first (3.14% at 30 wt% of light calcined magnesia) and then decreases (−3.23% at 50 wt% of light calcined magnesia). Unlike the sintering of dense materials, the volume change in porous aggregates due to calcination is influenced by a number of factors: firstly, the powder shrinks at high temperatures (the linear shrinkage is negative), which is one of the characteristics of the sintering process [22]; secondly, for the chemical reactions involved, the volume change caused by the phase change also needs to be considered (as mentioned previously, the formation of magnesia-alumina spinel results in some volume expansion); finally, although porous materials are not involved in the densification process at the end state of sintering, different sintering mechanisms will still have an impact on the initial and intermediate stages of sintering. Therefore, for the sample with 30% of light calcined magnesia, the volumetric expansion effect caused by the chemical reaction is dominant, so its line shrinkage is positive; for the samples with 40% and 50% of light calcined magnesia, the activated sintering mechanism caused by the chemical reaction plays a major role, so their line shrinkage is negative. Interestingly, as shown in Figure 7b, the apparent porosity of the samples does not change like the linear shrinkage and demonstrates a trend of first increasing and then decreasing. Specifically, the apparent porosity of the sample with 50% of light calcined magnesia decreases to 48.2% but is still higher than the blank sample of 47.5%. From Figure 7c, the bulk density of the samples decreases from 2.09 g·cm^−3^ of the blank sample to 1.74 g·cm^−3^ of the sample with 30% of light calcined magnesia and then increased to 2.04 g·cm^−3^ of the sample with 50% of light calcined magnesia. This suggests that the introduction of light calcined magnesia contributes to the lightweighting of aggregates.

#### 3.2.4. Cold Compressive Strength and Thermal Shock Resistance

**Figure 8:**
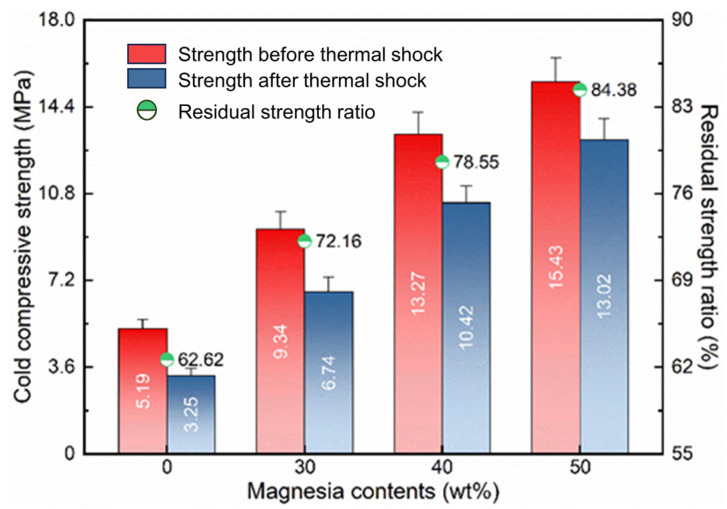
Cold compressive strength and thermal shock resistance of the samples with different amounts of light calcined magnesia after calcining at 1400 °C for 1 h.

Figure 8 shows the cold compressive strength and thermal shock resistance of the samples with different amounts of light calcined magnesia after firing at 1400 °C for 1 h. Compared with the blank sample (5.19 MPa and 3.25 MPa), both the directly measured cold compressive strength and the residual compressive strength after the quenching experiment of the additive-containing sample increase with the increase in the amount of light calcined magnesia. The increase in cold compressive strength is attributed to the formation of magnesium-alumina spinel which improves the degree of interfacial bonding of the samples and becomes more effective as its content increases. Similarly, the improvement in the thermal shock resistance of the samples is closely related to the formation of magnesia-alumina spinel, and the role of zirconia cannot be ignored. As shown in Figure 5, the crystal form of zirconia in the blank sample is the monoclinic system (low temperature type, m-ZrO_2_), while that in the samples containing additive is the tetragonal system (high-temperature type, t-ZrO_2_). In fact, phase transformation toughening is a common ceramic reinforcement method, which can effectively improve properties such as strength, fracture toughness, and thermal shock resistance [23]. Therefore, for the samples with light calcined magnesia, as the addition amount increases, more magnesia-alumina spinel phases are formed, and the linear expansion coefficient of the sample decreases; meanwhile, the zirconia in the samples can be stabilized into a tetragonal crystal form due to the occurrence of solid solution reaction. According to Hasselman’s theory [24,25,26], the thermal shock resistance parameters of the additive-containing samples is higher, so their thermal shock resistance is better.

### 3.3. Analysis of Lightweight Aggregate Formation

#### 3.3.1. Thermodynamics

Thermodynamic data are one of the effective means to analyze complex multi-phase systems. The relevant chemical reactions involved in this work are listed in Table 2. 

**Figure 9:**
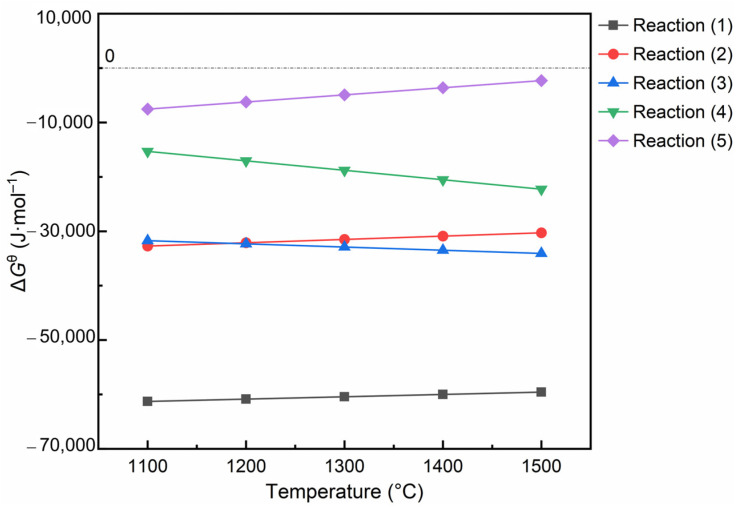
Relationship between Δ*G*^θ^ and temperature for related reactions.

From Figure 9, the corresponding Gibbs free energy, the values of all reactions are negative, meaning that all are possible. By further comparison, the Δ*G*^θ^ of reaction (1) is less than that of reaction (2) in the preset temperature interval, so the Mg_2_SiO_4_ phase is easier to form than MgSiO_3_. Similarly, due to the smaller Gibbs free energy of reaction (3), alumina is more inclined to react with magnesia to form the MgAl_2_O_4_ phase than to form 3Al_2_O_3_·2SiO_2_ or ZrSiO_4_. Therefore, in agreement with the experimental results, the samples maintained the same phases at different calcination temperatures.

#### 3.3.2. Lightweight Mechanism

From the data in Section 3.2, with the introduction of light calcined magnesia, the apparent porosity and mechanical properties of the samples can be simultaneously improved to a certain extent. This is a beneficial result caused by the phase change of the samples before and after calcination. In this case, the carbon element (flake graphite in used Al_2_O_3_-ZrO_2_-C refractories), which was not detected after calcination, acted as a pore former; meanwhile, the volume effect accompanying the phase change process is also an important contributor. For chemical reactions involving multiple phases, the volume change can be calculated using the following equation:

(6) $$∆V=\left((\sum \frac{M_{k}\times b_{k}}{\rho_{k}}-\sum \frac{M_{i}\times a_{i}}{\rho_{i}})/\sum \frac{M_{i}\times a_{i}}{\rho_{i}}\right)\times 100\%$$

There, *M_k_* and *M_i_* are the molar masses of the *k*-th product and the *i*-th reactant, respectively; *b_k_* and *a*_i_ are the balancing coefficients of the *k*-th product and the *i*-th reactant, respectively; and *ρ_k_* and *ρ_i_* are the theoretical densities of the *k*-th product and the *i*-th reactant, respectively [18].

**Figure 10:**
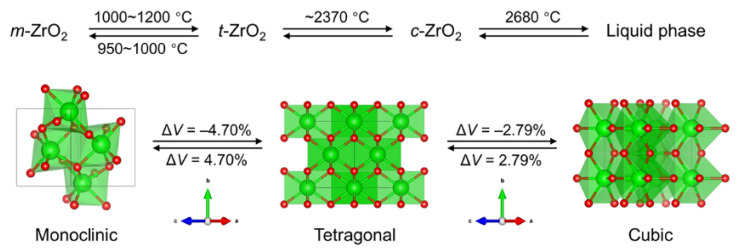
Different crystal forms of ZrO_2_.

Calculations show that the formation of Mg_2_SiO_4_ is accompanied by a volume contraction of 10.36%, while the formation of MgAl_2_O_4_ is accompanied by a volume expansion of 7.96%. Because the content of Al_2_O_3_ in the raw materials is much greater than that of SiO_2_, the content of MgAl_2_O_4_ in the sample after calcination is much greater than that of Mg_2_SiO_4_, which means that from the overall perspective, the volume of the samples is still expanded. In addition, as shown in Figure 10, ZrO_2_ undergoes crystal transformation at different temperatures, which is also accompanied by a certain volume change. Considering that it accounts for a small proportion in the raw material, its effect on the porosity of the samples can be ignored. On the other hand, this volume change is crucial to the strength and thermal shock resistance of the specimen, namely the phase transformation toughening effect.

## 4. Conclusions

In this work, lightweight MgO-MgAl_2_O_4_ composite aggregates with high strength and good thermal shock resistance were successfully prepared at 1400 °C for 1 h. The conclusions obtained are as follows:

(1) As the calcination temperature rises, the apparent porosity of the prepared lightweight aggregates decreases, and the cold compressive strength and thermal shock resistance increase. The comprehensive performance of the sample fired at 1400 °C is relatively balanced: the linear shrinkage is −0.25%; the apparent porosity is 51.1%; the bulk density is 1.92 g·cm^−3^; the cold compressive strength is 13.27 MPa; and the residual strength ratio (thermal shock resistance) is 78.55%.
(2) With the introduction of light calcined magnesia, the apparent porosity, cold compressive strength, and thermal shock resistance of the prepared lightweight aggregates are effectively improved. This can be attributed to the formation of the magnesia-alumina spinel and the phase transformation toughening mechanism. However, for applications in high-temperature insulation environments, the effects of properties such as thermal conductivity need to be further considered to determine the optimal addition amount.

## Figures and Tables

**Table 1:** Chemical composition of raw materials (wt%).

	Al_2_O_3_	CaO	C	MgO	SiO_2_	ZrO_2_	Other
Used Al_2_O_3_-ZrO_2_-C refractories	65.83	-	22.1	0.29	3.83	7.55	0.4
Light calcined magnesia	-	1.09	-	95.52	2.69	-	0.7

**Table 2:** Main chemical equations and corresponding Gibbs free energies.

Chemical Equation	Gibbs Free Energy	Serial Number
2MgO(s) + SiO_2_(s) = Mg_2_SiO_4_(s)	−68,200 + 4.31T (*T* < 2171.15 K)	(1)
MgO(s) + SiO_2_(s) = MgSiO_3_(s)	−41,100 + 6.1T (*T* < 1850.15 K)	(2)
MgO(s) + Al_2_O_3_(s) = MgAl_2_O_4_(s)	−23,604 − 5.91T (*T* < 1973.15 K)	(3)
3Al_2_O_3_(s) + 2SiO_2_(s) = 3Al_2_O_3_·2SiO_2_(s)	8600 − 17.41T (*T* < 2123.15 K)	(4)
ZrO_2_(s) + SiO_2_(s) = ZrSiO_4_(s)	−25,496 + 13.08T (*T* < 1949.15 K)	(5)

## Data Availability

Data are contained within the article.
